# Prototype of a Multimodal and Multichannel Electro-Physiological and General-Purpose Signal Capture System: Evaluation in Sleep-Research-like Scenario

**DOI:** 10.3390/s25092816

**Published:** 2025-04-30

**Authors:** Pablo Cevallos-Larrea, Leimer Guambaña-Calle, Danilo Andrés Molina-Vidal, Mathews Castillo-Guerrero, Aluizio d’Affonsêca Netto, Carlos Julio Tierra-Criollo

**Affiliations:** 1 Biomedical Engineering Research Group—GIIB, Universidad Politécnica Salesiana, Cuenca 010102, Ecuador; lguambana@est.ups.edu.ec; 2 Biomedical Engineering Program, Alberto Luiz Coimbra Institute for Graduate Studies and Research in Engineering (Coppe), Federal University of Rio de Janeiro, Rio de Janeiro 21941-914, Brazil; dmolinav@peb.ufrj.br (D.A.M.-V.); mathews@peb.ufrj.br (M.C.-G.); aluizionetto@peb.ufrj.br (A.d.N.); carjulio@peb.ufrj.br (C.J.T.-C.)

**Keywords:** multichannel biomedical signal acquisition system, multimodal physiological signals, ADS1299

## Abstract

The simultaneous analysis of electrophysiological signals from various physiological systems, such as the brain, skeletal muscles, and cardiac muscles, has become increasingly necessary in both clinical and research settings. However, acquiring multiple modalities of electrophysiological data often necessitates the use of diverse, specialized technological tools, which can complicate the establishment of a comprehensive multimodal experimental setup. This paper introduces a prototype system, named the Multimodal–Multichannel Acquisition Module—MADQ, designed for the simultaneous acquisition of multimodal and multichannel electrophysiological and general-purpose signals. The MADQ comprises three distinct capturing blocks, each equipped with separate reference circuits, supporting a total of up to 40 electrophysiological input channels, alongside 4 channels of analog input and 4 channels of digital input signal. The system is capable of sampling frequencies up to 16 kHz. Key features of the MADQ include individually configurable bipolar recording, lead-off detection capability, and real-time online filtering. The system’s functional performance was characterized through metrics such as Input-Referred Noise (IRN), Noise-Free Bits (NFB), and Effective Number of Bits (ENOB) across varying gain and sampling frequencies. Preliminary experiments, conducted in a setup emulating a sleep study with auditory evoked potential detection, demonstrate the system’s potential for integration into multimodal experimental scenarios.

## 1. Introduction

Electrophysiological signals play a crucial role in medical and research contexts, offering valuable insights into the functioning of diverse physiological systems and facilitating precise study and diagnosis [1,2]. For instance, electroencephalography (EEG) signals support the study and diagnosis of neurological disorders such as epilepsy, autism, neuropsychiatric disorders [3,4], and sleep disorders [5]. Likewise, muscle activity captured in electromyography (EMG) is essential in the evaluation of neuromuscular disorders and the planning of physical rehabilitation [6,7,8]. Electrocardiography (ECG) enables the diagnosis of cardiovascular diseases such as arrhythmias and cardiopathies [9]. Electro-oculography (EOG) also supports the diagnosis of sleep disorders such as sleep apnea [10].

In certain clinical and research contexts, it becomes necessary to study multiple physiological systems involved concurrently in an activity or medical condition. For instance, when investigating individuals with sleep disorders, EEG, EOG, and EMG signals are monitored simultaneously to comprehend the intricate patterns of brain, ocular, and muscular activity during sleep. Additionally, the study of neuromuscular diseases relies on combining EMG and EEG signals to offer a comprehensive understanding of neuromuscular function and the central nervous system, aiding in diagnostic and treatment tasks [11,12,13]. Recent research has employed simultaneous monitoring of ECG, EEG, and EMG in the study of neurodegenerative disorders, such as Parkinson’s and Alzheimer’s diseases [14,15], aiming to gain profound insights into disease progression and to open perspectives in the development of more precise therapeutic strategies. These varied experimental setups may also incorporate signals beyond the electrophysiological nature, such as those from temperature, pressure, and acceleration sensors, among others.

Establishing experimental setups encompassing a wide variety of types of electro-physiological signals (i.e., the multimode signal approach), alongside signals from external sensors, may represent a technological challenge for many researchers. This situation may compel researchers to include multiple devices in an experimental setup which could complicate the experiment. Conversely, incorporating a multimode, multisignal platform may represent a highly specialized and costly technology that is challenging to adopt and find. Although some commercial platforms for multimode signal acquisition exist—such as the IXTA-220 or IXRA-834 by iWorx [16], the BrainVision Recorder by Brain Products [17], the MP160 by Biopac [18], and Nautilus PRO by g.tec medical engineering GmbH [19]—they exhibit limitations in specialized research protocols. First, these platforms often restrict access to custom configuration of acquisition parameters, as they are primarily designed for educational purposes and typically limit users to predefined experimental exercises. Second, these platforms typically lack the incorporation of multiple acquisition blocks, each equipped with independent reference circuits within a single device. This limitation restricts the concurrent and configurable capture of multiple channels of various types of physiological signals, such as ECG, EEG, and EMG, which traditionally require distinct reference points for adequate signal acquisition. Third, most commercial platforms utilize proprietary and closed-source software, which may obstruct the customization or integration of user-defined data-processing functions [20,21] or hinder the online monitoring of raw data. Research-grade platforms for multichannel electrophysiological signal acquisition in the literature [22,23,24] possess varying levels of hardware customization but often lack integrated multimodal support, support for a high number of recording channels, or comprehensive documentation for reproducibility and scalability. For example, ref. [22] describes a low-cost 4-channel wireless EEG unit for BCI applications (*f_s_* up to 2 kHz), limited to EEG/ECG without general-purpose signals integration. In [23], a portable 8-channel EEG system using ADS1299 and flexible dry electrodes (Ag NWs/PDMS) shows potential for future ECG/EMG use but lacks native multimodal capabilities and open-source firmware. Similarly, ref. [24] presents an 8-channel EEG system using ADS1299 and a PIC32 microcontroller (1 kHz, IRN < 0.5 µV RMS), restricted to EEG with minimal support for other bio-signals and limited open-source software.

This article presents the design, implementation, and evaluation of a prototype system named the Multimodal–Multichannel Acquisition Module—MADQ, which incorporates a configurable up-to-40-channel electrophysiological signal capture capability alongside an 8-channel general-purpose signal capture function, as well as configurable sampling frequency. The system incorporates an innovative architecture to support multimodal physiological signal acquisition through the integration of three independently referenced circuit blocks. A user application implemented in the widely research-and-academic-compatible software LabVIEW enables access to all information and functions in the system. The authors evaluated the performance of the system and tested its capability for capturing multimode signals in an experimental scenario emulating the multimode requirements of a sleep study [25,26,27].

## 2. Materials and Methods

### 2.1. Design

**Figure 1:**
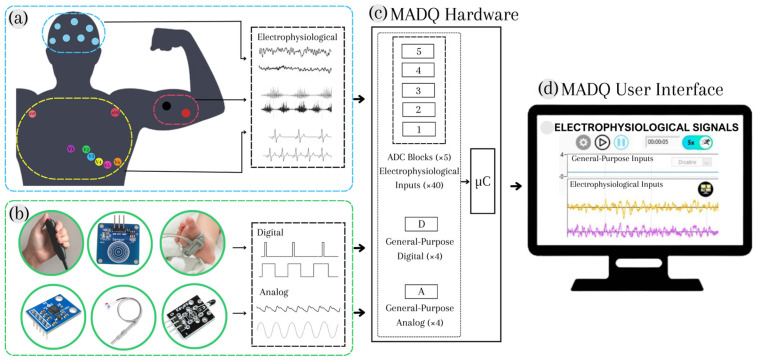
General block diagram of the MADQ. (**a**) Multimode electrophysiological signals; (**b**) General-Purpose (GP) digital and analog signals; (**c**) hardware component to capture signals; (**d**) User Interface (UI) that enables configuration and management of the acquisition functions.

The design process was conducted using a System Modelling Language (SysML) paradigm [28], utilizing Visual Paradigm Software v17.1, which allows for the construction of blocks, activities, use cases, and requirement diagrams to specify functionalities, performance, and safety requirements. The inclusion of SysML resources is evidenced through the following sections. Figure 1 presents a general block diagram of the MADQ, illustrating its two primary components. A hardware component (Figure 1c) oversees the capturing of multichannel/multimode electrophysiological (Figure 1a) and general-purpose analog/digital signals (Figure 1b) in an experimental scenario. The hardware communicates this information via Ethernet connection (UDP protocol) to the software component: a User Interface (UI) implemented in a computer that enables an operator to configure and manage an experimental protocol (Figure 1d).

#### 2.1.1. Hardware

**Figure 2:**
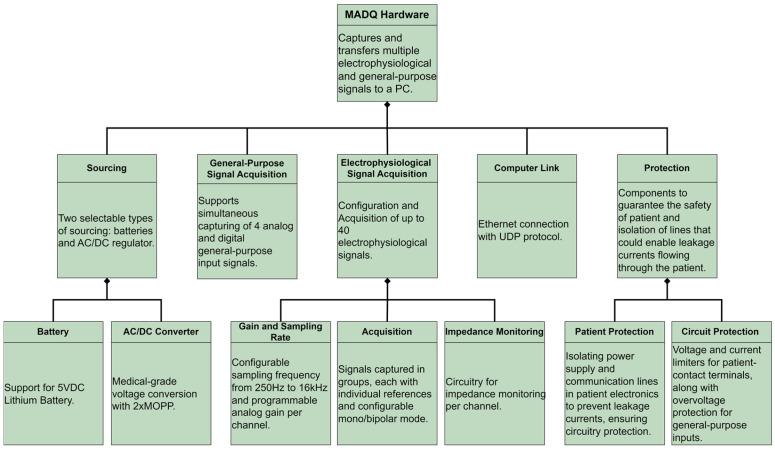
Requirement diagram of hardware.

Following the SysML strategy, this study initiated the hardware design process by developing a comprehensive requirement diagram (Figure 2). This diagram delineates core functionalities, performance metrics, and security parameters essential for ensuring hardware compatible with regulatory standards required for a medical device [29,30,31].

**Figure 3:**
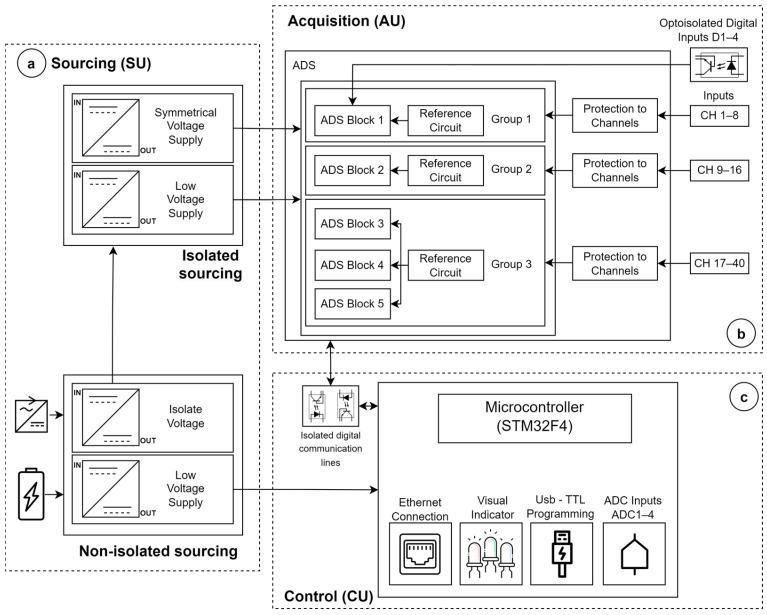
Block diagram of hardware architecture illustrating sourcing SU (**a**), acquisition AU (**b**), and control CU (**c**) units.

To fulfill the requirement diagram, the hardware architecture integrates three units (Figure 3): the sourcing unit (SU), control unit (CU), and signal acquisition unit (AU). The SU (Figure 3a) accepts input voltages from either a medical-grade AC/DC voltage regulator (jack-type connector) or an external 5 VDC battery (USB mini-B port). The AC/DC regulator must comply with input/output isolation compatible with two means of patient protection (2xMOPP, IEC60601) and EMI/EMC compatibility (e.g., the model SDM24-UD/UCI/INC with 4 kVAC isolation voltage and 0.1 mA leakage current—Mean Well Enterprises/MW, New Taipei, Taiwan). The USB mini-B port also enables the uploading of the microcontroller’s firmware through a USB-to-serial converter (CH340C—Nanjing QinHeng Corp./WCH, Nanjing, China). A first +3.3 VDC voltage regulator (LM1117MP-3.3—Texas Instruments Incorporated/TI, Dallas, TX, USA) powers the electronic components of the CU, ensuring that this power line remains separate from the patient line. Furthermore, an isolated DC–DC voltage regulator (PWR1303AC with 2xMOPP, 8 kV insulation, and max. leakage current of 2 μA—Advanced Energy Industries/AE, Inc., Denver, CO, USA) energizes the AU, providing electrical protection to the front-end electronic circuits (applied part of the equipment) from the connection line. This regulator serves as an additional layer of protection in the event of failure (isolation breakdown) of the general power supply. The isolated DC–DC outputs are regulated at voltages of ±2.5 and +3.0 VDC, ultimately powering the analog and digital circuits of the AU.

The AU (Figure 3b) comprises five blocks dedicated to Analog-to-Digital Conversion (ADC). Each block utilizes the 24-bit resolution ADS1299 (Texas Instruments Incorporated/TI, Dallas, TX, USA) [32] that supports up to eight bipolar inputs for electrophysiological signals, four digital inputs, sampling frequencies between 0.25 and 16 kHz, and programmable gains (PGA) individually by channels adjustable from 1 to 24. The five acquisition blocks are organized into three groups, with each group having a distinct reference circuit. ADS block 1 (Group 1, channels 1–8) and ADS block 2 (Group 2, channels 9–16) possess individual reference circuits, while ADS blocks 3, 4, and 5 (Group 3, channels 17–40) share a common reference circuit (Figure 3b). Individual references separated by groups of ADS blocks allow simultaneous capture of diverse signal modalities. To streamline the physical connectors within the device chassis, only block 1 supports configurable monopolar/bipolar acquisition, while blocks 2 to 5 exclusively facilitate monopolar acquisition. All input channels incorporate overvoltage protection circuits using Schottky diodes (BAT54S—Diodes Incorporated/DI, Plano, TX, USA). The ADS1299 chip itself incorporates electrostatic discharge protection compliant with the human body model (HBM, ANSI/ESDA/JEDEC JS-001 of ±1000 V) and charged device model (CDM, JEDEC JESD22-C101 ±500 V) [32]. Moreover, the design incorporates four General Purpose (GP) digital inputs (embedded in ADS block 1) to enable synchronous event recording from external devices or sensors, which is a valuable feature in event-relate potential applications [33,34,35]. Each digital input incorporates optocoupler circuits (PS2501A—Toshiba, Tokyo, Japan) to ensure isolation.

The CU (Figure 3c) is based on the 32-bit ARM Cortex-M4 MCU (STM32F407—STMicroelectronics/ST, Geneva, Switzerland), clocked at 168 MHz, which establishes connections to the computer via a 10/100 Mbps Ethernet interface (LAN8720AI-CP—Microchip Technology Inc., Chandler, AZ, USA). The MCU interfaces with five ADS blocks in the AU through a standard SPI protocol, employing five distinct chip select signals and common control lines for functions such as reset, start, and data-ready [32]. Data access (read/write) to ADS blocks by the CU occurs through a multiplexing scheme. The ADS-Block-1 data-ready line notifies the CU when data are available for retrieval. To enhance isolation of the patient line, digital communication between the CU and AU utilizes optocouplers (ISO7741—Texas Instruments Incorporated/TI, Dallas, TX, USA). In addition, the CU features four general-purpose analog inputs ranging from 0 to +3.3 VDC, utilizing the MCU’s 10-bit ADCs and incorporating overvoltage protection circuits and conditioning circuitry. The firmware algorithm of the MCU, developed using the STM32Cube IDE v1.11.0 platform, performs two primary functions: (1) configuring the ADC blocks in response to commands from the UI, and (2) synchronously acquiring data from all ADS blocks as well as general-purpose analog signals, with periodic transfers to the PC.

#### 2.1.2. Software

**Figure 4:**
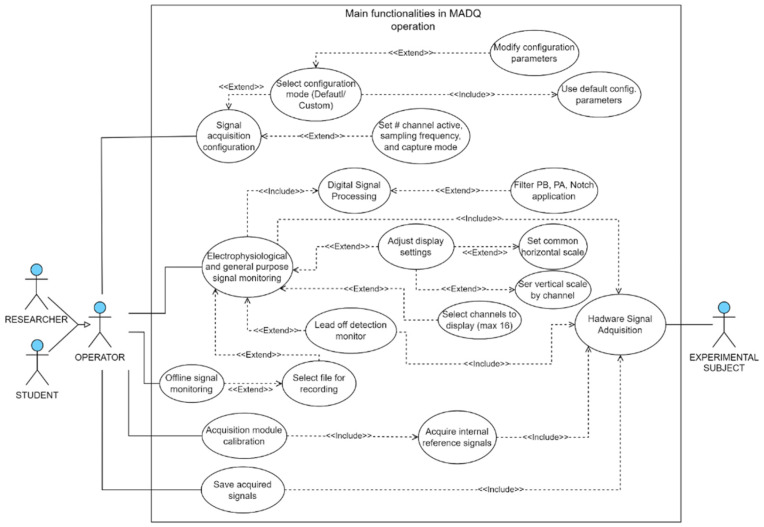
Use case diagram illustrating main functionalities in MADQ operation. In the diagram, the “Extend” relationship represents an optional process, whereas the “Include” relationship represents a mandatory process that is executed alongside its associated master process.

The design of the UI started with the creation of a use case diagram outlining the primary functionalities available to the user (Figure 4). The UI was developed using LabVIEW v21.0 [36], software widely embraced in academic and research settings [37,38,39]. The UI incorporates four main functionalities: (1) **acquisition configuration**—enables users to set parameters such as the number of active input channels (electrophysiological and general-purpose), sampling frequency, gain, mode configuration (monopolar/bipolar), and enables impedance monitoring by channel; (2) **signal monitoring**—the core algorithm retrieves data from hardware, applies processing algorithms (including filters when enabled), and presents signals in real time according to configured scales. This feature enables impedance monitoring at the electrode contact site; (3) **data storage and data playback**—users can record a binary file containing all acquisition information, insert marks, and access the file for offline data visualization, and (4) **calibration**—this functionality performs acquisition of internal reference signals to adjust differences in offset and gain between channels.

### 2.2. Functional Tests

The evaluation of the MADQ’s functional performance was conducted in two stages. The first stage involved determining the maximum number of electrophysiological and general-purpose signal channels that could be simultaneously acquired relative to the sampling frequency *f_s_*. This was achieved by assessing the integrity of the internal reference signals provided by ADS1299 in 1 min recordings across various channel quantities (8, 16, 24, 32, 40) and *f_s_* ranging from 0.25 to 16 kHz, while also verifying the continuity of the digital frame numbers received from the hardware. In the second stage, performance parameters related to the intrinsic noise level and resolution of the ADS blocks were measured. These parameters included Input-Referred Noise (IRF), Noise-Free Bits (NFB), Effective Number of Bits (ENOB), and Offset Error. The calculations of these parameters were performed in accordance with the recommendations in [32], with all input channels shorted to internal ground. For this test, signals were recorded under two conditions: (1) a 40-channel, 20-second recording at varying *f_s_* of 0.25, 0.5, 1.0, 2.0 kHz, with gains of 1, 4, 12, 24 and; (2) an 8-channel, 20-second recording at varying *f_s_* of 4.0, 8.0, 16 kHz, with gains of 1, 4, 12, 24. The values of IRN, NFB, and ENOB were calculated as mean values for all channels under each condition.

### 2.3. Preliminary Experimental Test

The test is designed as an initial evaluation of the proposed technology; therefore, it did not encompass large cohorts of either healthy individuals or patients. The primary objective of this test is to assess the MADQ’s capability to concurrently capture multimodal signals from diverse sources. In this regard, the study implemented an experimental setup that simulates a sleep research environment, including both electrophysiological and non-electrophysiological analog and digital signals [5,12,13,25,26]. Three healthy individuals (2 males, 1 female), aged between 18 and 35 years, participated in the experiment conducted in the Signal and Image Processing Laboratory of the Biomedical Engineering Program of the Federal University of Rio de Janeiro (LAPIS/PEB/UFRJ), all providing informed consent prior to participation. The experimental procedures involving human subjects were conducted according to the approved protocol by the Ethics Committee of the University Hospital Clementino Fraga Filho of the Federal University of Rio de Janeiro (HUCFF/UFRJ), Brazil, under certificate number CAAE: 01143318.6.0000.5257. Participants were instructed to remove all jewelry from their necks, wrists, and ankles, settling in a reclining chair for signal recording.

**Figure 5:**
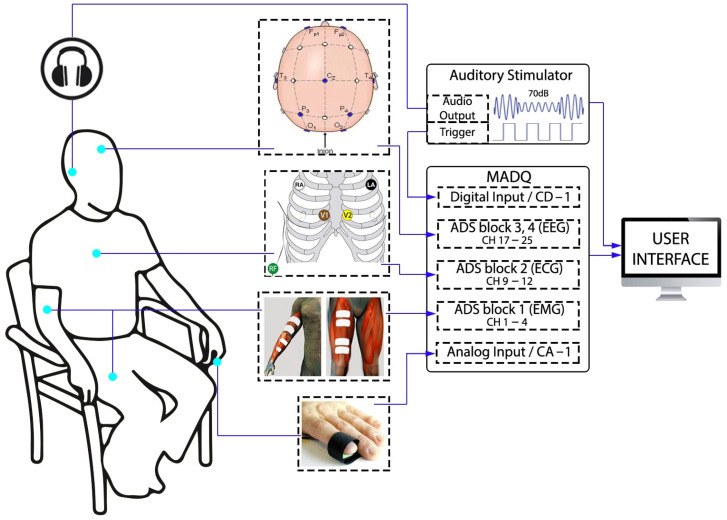
Experimental setup using the MADQ to capture electrophysiological and non-electrophysiological signals.

The experimental setup (Figure 5) incorporates three types of electrophysiological signals, and two general-purpose signals registered by the MADQ. The electrophysiological signals include the following: (i) Four bipolar EMG channels connected to ADS block 1, with two channels placed on the left forearm approximately halfway along the length of the biceps brachii and flexor digitorum superficialis muscles, and two channels along the left leg on the rectus femoris muscle. (ii) Four monopolar ECG channels connected to ADS block 2, with active electrodes placed just below the right and left clavicles and in the intercostal locations of V1 and V2. The reference electrode for ECG recording was on the right ankle and connected to the reference input of ADS block 2. (iii) Nine monopolar EEG channels connected to ADS blocks 3 and 4, with active electrodes placed according to the 10–20 system at O1, O2, P3, P4, T3, T4, Fp1, Fp2, Inion (just below the hairline). The reference electrode for the EEG recording was located at Cz and connected to the reference input of the MADQ corresponding to ADS blocks 3–5, and the ground (GND) electrode located was placed 2 cm from Fpz. A non-electrophysiological pulse plethysmograph analog signal provided by an PTN-104 sensor (iWorx Systems, Inc., Dover, NH, USA) [40] placed on the index finger of the left hand was directed to one of the GP analog inputs of the MADQ (ChA. #1). The signal from PTN-104 was first conditioned through a battery-powered instrumented amplifier circuit added externally (AD620 Microvolt/Millivolt Voltage Amplifier Module—Analog Devices Inc., Wilmington, MA, USA). Additionally, the experiment incorporated components for detecting an auditory evoked potential, specifically a steady-state auditory evoked potential ASSR [33], to assess the capability of the MADQ to capture signals synchronized with external events. In this regard, an external auditory stimulator device [34] was programmed to deliver a continuously modulated auditory stimulus in both ears, with sound intensity equivalent to 70 dB SPL. The combination of carrier and modulating frequencies by ear were 500 Hz and 31 Hz, respectively, for the left ear, and 2.0 kHz and 39 Hz, respectively, for the right ear. The stimulus-synchronized digital signal provided by the stimulator is recorded in one of the GP digital inputs of the MADQ (ChD. #1). All devices and components involved in the experimental scenario were powered using a unique external battery module (BP-95—PowerExtra, Shenzhen, China).

Data recording was conducted at *f_s_* of 1 kHz, with analog gains set at 1 for ECG, 6 for EMG, and 12 for EEG. Considering that the higher gain levels resulted in reduced IRN (as confirmed during functional testing), these levels were assigned to signals with lower magnitude. Prior to electrode placement, the regions underwent thorough cleaning with alcohol swabs. EMG and ECG recordings used Ag/AgCl disposable electrodes, while EEG used an electrode cap and conductive solution. Before recording, the impedance monitor in the MADQ was used to verify an electrode–skin interface impedance lower than 5 kHz.

**Figure 6:**
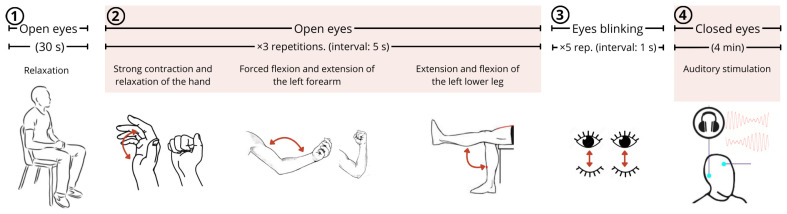
Sequence of events executed by participants in the experimental protocol.

Each participant was instructed to perform a sequence of actions intended to elicit characteristic changes in electrophysiological signals (Figure 6), as follows:

1. Maintaining muscle relaxation with eyes open (at rest) for thirty seconds;
2. Performing three repetitions of specific muscular movements with five-second interval: (i) contraction and relaxation of the hand (gripping and releasing), (ii) flexion and extension of the left forearm, (iii) extension and flexion of the left lower leg;
3. Blinking eyes five times with 1 s intervals;
4. Closing the eyes and maintaining a resting state for 4 min while being subjected to auditory stimulation.

Verbal commands signaled transitions through steps 1 to 4. Participants underwent a training stage before signal recording commenced.

The recorded signals underwent processing using Matlab (R2022b, 9.13.0.2320565). Specifically, ECG, EMG and EEG signals were subjected to bandpass filtering within the following ranges: 0.5 to 45 Hz for ECG, 10 and 500 Hz for EMG, and 0.5 to 50 Hz for EEG. To identify the evoked potential ASSR the EEG segments, delimited by the trigger signal (totaling 16 segments in a stimulation session, each lasting 16.034 s), underwent a temporal averaging and subsequently, a discrete Fourier transform. This approach aligns with established practices for detecting ASSR in the literature [26,33,34].

## 3. Results

**Figure 7:**
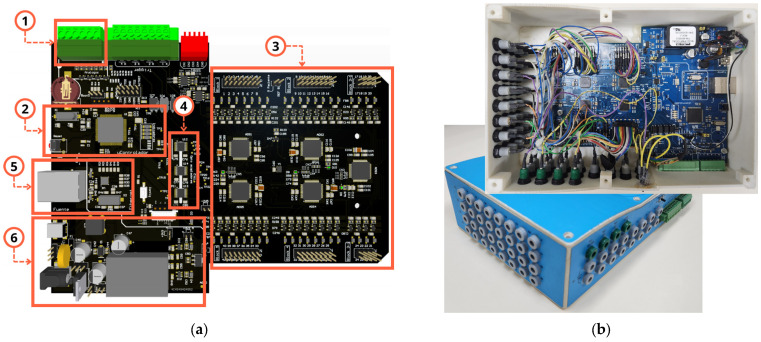
Hardware implementation and chassis. (**a**) Main components in PCB of the MADQ: analog GP inputs (1), CU (2), AU (3), isolation between AU and CU (4), ethernet (5), SU (6). (**b**) Internal and external views of the chassis highlighting external connector and circuitry implementation.

Figure 7a shows the 3D view of the main printed circuit board (PCB) of the MADQ’s hardware highlighting the components corresponding to the sourcing (SU), control (CU), and signal acquisition (AU) units. Figure 7b shows an external view of the chassis built by 3D printing using PLA (polylactic acid).

**Figure 8:**
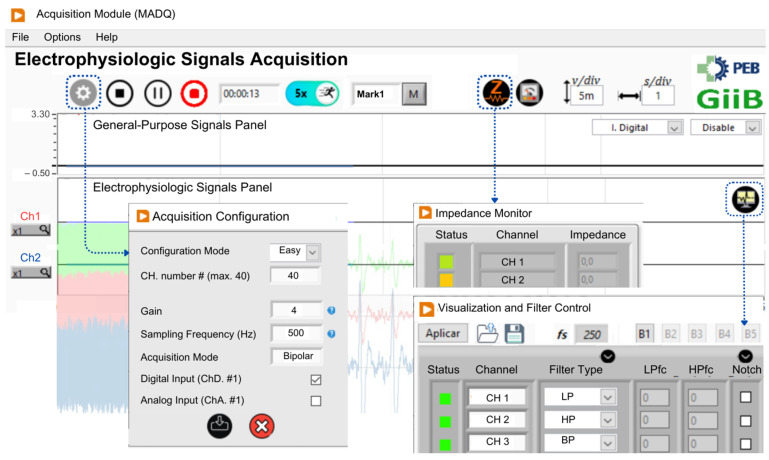
The LabVIEW-developed User Interface (UI). Excerpts of the main panels Acquisition Configuration, Visualization and Filter Control, and Impedance Monitor are shown.

Figure 8 displays the representative panels of the UI application for the MADQ, which are accessible to users. The **Acquisition Configuration** panel enables the adjustment of recording parameters, offering both easy and advanced views tailored to students or advanced researchers. The main visualization panel (**Acquisition Module**) incorporates two fields to separately visualize electrophysiological and GP signals with specific controls to set the vertical scale both individually per channel and globally, facilitating the visualization of signals having different magnitude orders. Also, the user can select the specific GP input to visualize. The **Visualization and Filter Control** panel allows users to configure simultaneous visualization of up to 16 signals from a maximum of 40 active channels, while also providing controls to enable online configurable lowpass, highpass, bandpass, and notch filtering. Under signal capturing, users can save signals (enabling a mark registration function) and access the **Impedance Monitor** panel to check electrode–skin interface impedance (available if previously activated). This last panel offers real-time feedback through a color-coded impedance indicator: green for impedances below 10 kΩ, yellow for values between 10 kΩ and 15 kΩ, and red for impedances exceeding 15 kΩ. This intuitive visual system enables users to quickly assess and optimize signal quality during the preparation phase. The usability and functionality of the controls implemented in the UI, as well as the overall operability of the system, were refined through an iterative process involving testing, feedback collection, and subsequent adjustments. This refinement process was conducted over multiple usability sessions with researchers (*n* = 10) and students (*n* = 120) from universities in Ecuador (UPS) and Brazil (UFRJ) between January 2021 and January 2024.

### 3.1. Functional Evaluation

Table 1 summarizes the results of testing the MADQ’s capacity for capturing a maximum number of channels at different *f_s_*. For *f_s_* ≤ 1 kHz (including 0.25 and 0.5 kHz), the MADQ can capture up to 40 electrophysiological channels (EC), along with 4 analog and 4 digital GP channels. However, as *f_s_* increases beyond 1 kHz, the number of channels the MADQ can capture decreases as follows: at 2 kHz, the maximum number of EC drops to 32, with 3 analog GP channels; at 4 kHz, it can capture 24 EC and 2 analog GP channels; at 8 kHz, it can capture 16 EC and 2 analog GP channels; and at 16 kHz, 8 EC and 1 analog GP channel. Importantly, the MADQ captures all the 4 digital GP inputs even at the highest sampling frequency of 16 kHz.

**Figure 9:**
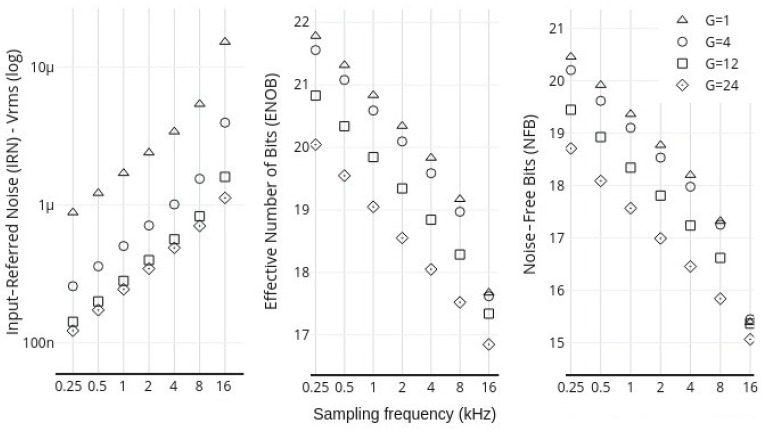
Mean IRN, ENOB, and NFB for the MADQ at different *f_s_* and gain values.

Figure 9 presents the behavior of IRN, NFB, and ENOB (ER) parameters for the MADQ. Each point in the graph represents the average parameter value across all active channels in the test: 40 channels for *f_s_* lower than 2 kHz and 8 channels for *f_s_* higher than 4 kHz. The acquisition performance parameters of the MADQ show a clear correlation with both gain and *f_s_*. When examining the relationship with gain, increasing the gain results in a lower Input-Referred Noise (IRN) but also a decreased Signal-to-Noise Ratio (SNR). This is primarily due to the reduced dynamic input range, which negatively impacts the performance parameters of Noise-Free Bits (NFB) and Effective Number of Bits (ENOB). For instance, at a typical *f_s_* 1 kHz, the IRN, ENOB, and NFB are 1.7 µV RMS, 19 bits, and 20 bits, respectively. However, when *f_s_* is increased to 16 kHz, these values change to 15.2 µV RMS, 17 bits, and 15 bits, respectively.

### 3.2. Experimental Evaluation

**Figure 10:**
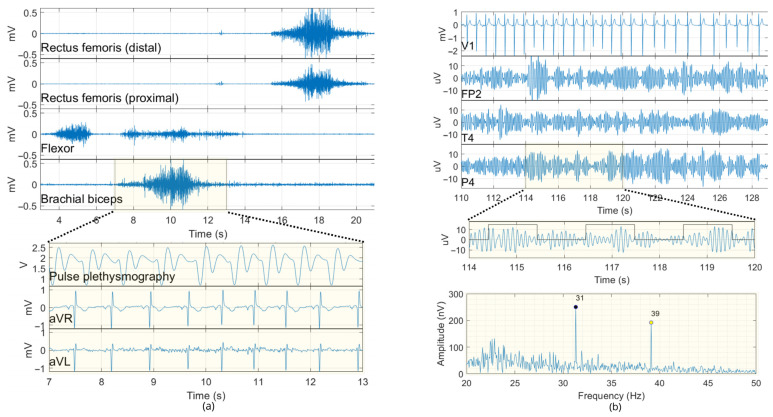
Extract of simultaneous recorded signals during a multimodal signal-acquisition scenario. (**a**) EMG (10–500 Hz), ECG (0.5–45 Hz), and pulse plethysmography signals. (**b**) EEG (1–50 Hz) derivations Fp2, T4, P4 alongside ECG derivation (V1) and stimulus-synchronized digital signals. The spectrum of the P4 derivation (filtered between 20 and 50 Hz) highlights ASSR responses.

Figure 10 displays an excerpt of processed signals from volunteer #1 during the multimode signal acquisition session. In Figure 10a, the activity of four EMG channels is shown during one of three sequences of muscular movements. This figure also highlights the concurrent capture of other signals alongside the EMG, specifically the aVR and aVL derivations of ECG, as well as the pulse plethysmography signal (analog GP input). All these signals correspond to the period when there was no auditory stimulation (open-eyes period). Figure 10b displays the EEG derivations (Fp2, T4, P4) recorded during auditory stimulation (closed-eyes period) along with the ECG derivation V1. This figure also includes a segment of the simultaneously recorded stimulus-synchronized digital GP signal (trigger signal), which is presented in conjunction with the corresponding signal from derivation P4. The FFT of the signal in P4 reveals ASSR responses at modulation frequencies 31 and 39 Hz, with amplitudes of 250 and 192 nV, respectively. The characteristic amplitudes for the different electrophysiological signals recorded were averaged across volunteers. For the EMG signals, the rms values during muscle contraction were approximately 87 µV RMS (range: 48 to 133) for the flexor, 140 µV RMS (range: 78 to 272) for the brachial biceps, and 58 and 79 µV RMS (range: 26 to 126) for the upper and lower femoral muscles, respectively. The average resting potential for EMG was 2 µV RMS. For the ECG, the QRS had mean amplitudes ranging from 1.8 to 2.5 mVpp across all derivations (aVR, aVL, V1, V2). The EEG signals exhibited a rms value of 5.29 µV.

**Figure 11:**
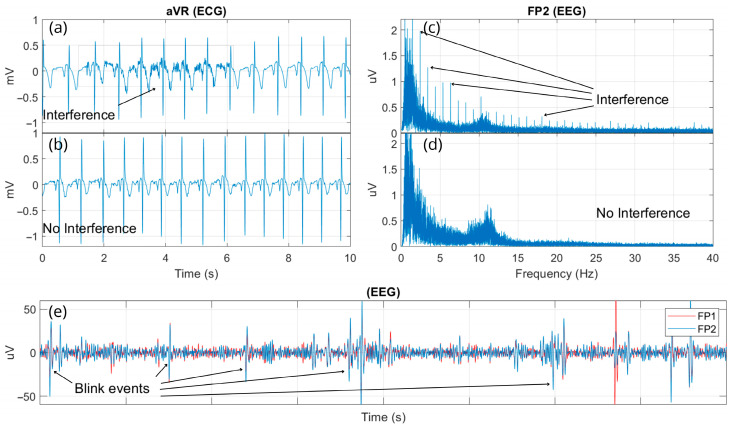
Illustration of interference events encountered during the experiment preparation phase for multimodal signal acquisition. (**a**) EMG interference affecting ECG recordings; (**b**) ECG signal without EMG interference after repositioning reference electrode; (**c**) Harmonics of trigger signal interference observed in the EEG spectrum due to improper coupling and lack of ground shielding; (**d**) EEG spectrum after resolving trigger signal interference; and (**e**) Example of common EEG interference caused by blinking.

This work also presents some interference events (Figure 11) encountered during the pre-experimental phase of multimodal signal acquisition sessions (experiment preparation), which may serve as a guide for researchers and students using the MADQ. In Figure 11a, an EMG activity originating from the femoral/biceps muscles is identifiable, overlapping and appearing in the ECG waveform recording. This interference occurred when the reference electrode for Block 1 was initially positioned on the carpal bone. Upon repositioning this electrode on the iliac crest bone, a signal with no interference was recorded, as shown in Figure 11b. Additionally, Figure 11c presents a sample of the EEG spectrum recorded at the Fp2 electrode, which was affected by interference from the stimulus-synchronized digital signal delivered from the auditory stimulator. This interference on EEG was attributed to two factors: improper coupling of EEG electrodes during recording and the absence of ground shielding in the connection of the trigger signal from the auditory stimulator to the MADQ. Figure 11d demonstrates the spectrum from the same EEG electrode after the interference factors were resolved, showing no evidence of interference. Finally, Figure 11e illustrates a common interference event in EEG recordings, caused by blinking or arm movements.

## 4. Discussion

This study has presented the design, implementation, and both technical and experimental evaluation of a prototype of multichannel electro-physiological and general-purpose signal acquisition system, named the MADQ. The MADQ showed the capacity to be integrated into scenarios involving various types of physiological signals with differing magnitudes and frequency parameters. The authors identified that using SysML in the design phase to build graphical models contributed to establishing system requirements, use cases, and functionalities, significantly improving the iterative design → test → feedback process to achieve the specifications of the MADQ. This experience is compatible with other studies utilizing SysML [41,42]. The authors observed an agile prototyping process when allocating extended periods of time to the requirement-gathering stage and including a multidisciplinary approach when building diagrams [43].

The MADQ system utilizes its hardware capabilities for signal acquisition, supporting up to 40 electrophysiological channels, 4 digital inputs, and 4 analog inputs when *f_s_* ranges between 250 Hz and 2 kHz. However, as the *f_s_* increases, the system’s capacity decreases: the number of available electrophysiological channels is reduced to 32 at 4 kHz, 16 at 8 kHz, and only 8 at 16 kHz. Similarly, the number of GP analog inputs decreases from 4 to 1 at 16 kHz. This reduction is attributable to the proposed hardware architecture and the time required to multiplex readings from up to five ADS blocks and the ADC peripheral (analog GP inputs). In the current design, the micro-controller (and its associated firmware) requires approximately 30 μs to capture data from each ADS block, amounting to a total of 150 μs for all blocks, with an additional 10 us per each ADC channel enabled. Achieving a 40-channel reading at 16 kHz would require completing each sampling cycle in less than 62.5 μs. While FPGA-based preprocessing schemes [44,45] could enable such high-speed multichannel operation, this capability offers limited practical value for the MADQ’s intended applications. This rationale stems from two considerations: (1) conventional electrophysiological studies predominantly involve multi-channel acquisition of low-frequency biopotential, while (2) specialized high-frequency protocols (e.g., invasive EMG, auditory evoked potentials like BERA [35,46,47] typically utilize only a limited number of channels. Consequently, the MADQ provides sufficient bandwidth for some specialized scenarios (8 channels, *f_s_*: 16 kHz) and enables capturing of a higher number of channels for conventional biosignals (40 channels, *f_s_*: 2 kHz or lower). In future studies, the MADQ system may incorporate a flexible acquisition scheme—both in software and hardware—that enables variable sampling rates for each ADS block. These rates would be adapted according to the Nyquist frequencies of target electrophysiological signals, thereby optimizing memory use and increasing the maximum number of channels that can be simultaneously recorded.

The levels of IRN, NFB, and ENOB observed in this study are comparable to those reported in similar works utilizing the ADS1299 IC [32,48]. The minimum IRN levels achieved meet the noise requirements for various electrophysiological signals, such as EEG [49,50], EMG, ECG, and EOG [51,52,53,54,55]. However, it is important for users to consider that selecting higher gain values for a particular channel, while it can reduce the IRN, also decreases input dynamic range. This trade-off is important to consider, as a reduced dynamic range increases the risk of saturating the input stage in the presence of non-stationary potentials [56]. The lowest observed ENOB and NFB values were 17 bits and 15 bits, respectively, which are sufficient to enable the acquisition of a wide range of biomedical signals. This includes, for example, the identification of ASSR at 31 Hz with amplitudes as low as 192 nV (Figure 10b). Future work will focus on exploring analog-to-digital conversion technologies that offer improved performance in terms of signal-to-noise ratio (SNR) and gain.

Additionally, since the MADQ system uses a single *f_s_* across all ADS blocks, the user should select the lowest possible *f_s_*. This not only enhances the IRN, NFB, and ENOB parameters but also reduces the data recording size. Users dealing with extended recording sessions should consider appropriate data storage strategies to maintain stable system performance. The decrease in NFB values observed at higher sampling frequencies is primarily attributed to the delta–sigma architecture of the ADS1299 used in the MADQ system, which reduces the efficiency of the internal digital filters as the sampling rate increases, thereby reducing the system’s capacity to suppress noise. To manage noise levels, the MADQ design incorporates components that ensure power supply stability and reduce external noise sources, supported by an optimized PCB layout, proper grounding, and the use of low-noise analog front-end elements. At the experimental level, maintaining good electrode–skin contact and using matched differential signal paths is recommended to minimize input impedance mismatch and cable-related artifacts.

Table 2 presents a comparative analysis of operational parameters between the MADQ system and representative commercial/research-grade acquisition platforms. The MADQ demonstrates competitive performance through three key characteristics: (1) configurable channel capacity (8–40 channels), (2) adjustable sampling frequency (0.25 to 16 kHz), and (3) low noise floor (0.24–1.7 µV RMS). These specifications position the MADQ favorably against established systems such as the OpenBCI Cyton (8 channels, 250 Hz, <1 µV RMS) [57] and the g.USBamp (16 channels, 38.4 kHz, <0.2 µV RMS) [58], while approaching the capabilities of specialized systems like the NeXus Q32 (32 channels, 4 kHz, <0.8 µV RMS) [59].

The LabVIEW-developed user interface meets all established requirements and is based on feedback from a broad group of preliminary users; it is deemed suitable for academic and research applications. However, certain technical characteristics should be considered when applying the MADQ system in real-time data acquisition scenarios. The MADQ uses a packet-based communication scheme, consisting of sample groups ranging from 2 to 20, depending on the sampling frequency (only for 2 kHz or higher) and whether more than two ADS blocks are active (i.e., more than 16 channels). The packets are temporarily buffered in hardware before transmission. While this approach enables efficient use of the communication channel, it may introduce variable latency that could impact applications requiring high-speed real-time feedback or closed-loop control. In addition, acquisition setups involving high data volume increase the computational load on the host computer for tasks in software such as data filtering, signal conditioning, and real-time visualization. In our tests, no significant delays were observed when using a quad-core processor, 16 GB RAM terminal.

The MADQ further differentiates itself through its research-focused design, which offers two distinctive features: a modular architecture with independent signal acquisition blocks that enable true multimodal operation, and isolated reference configurations and full customization capabilities for seamless integration into diverse experimental paradigms. However, two current limitations should be noted: the system lacks medical certification, which may restrict its immediate application in certain clinical research settings (such as a Critical Care Unit), and wireless functionality has not yet been implemented, potentially limiting flexibility in some experimental setups. In addition, the current version of the MADQ provides bipolar recording capability only in Blocks 1 and 2. However, this capability can be extended to other blocks with minimal modifications, including an increase in chassis dimensions.

Tests emulating a sleep study scenario demonstrated the MADQ system’s capability to independently record three signal modalities—EEG, EMG, and ECG—using separate reference circuits. These results highlight the system’s ability to be integrated into experimental setups requiring multimodal acquisition of signals with varying sources, amplitudes, frequencies, and noise characteristics. The experiments also confirmed the feasibility of incorporating external devices connected to the analog and digital GP inputs, making the system suitable for scenarios requiring signal synchronization. The recorded electrophysiological signals displayed amplitudes consistent with standard values [51,52,53]. The limited sample size and the use of a simulated scenario in the validation phase of this study may constrain the representativeness of the results. The system’s performance and effectiveness still require validation through future studies conducted in broader and more realistic settings. These studies should include a wider range of physiological and clinical conditions, exposing the system to the specific challenges of each context and enabling a more comprehensive evaluation of its performance and usefulness.

Electromagnetic interference was observed under conditions such as electrode decoupling, wiring proximity, and the volume conductor effect. To mitigate noise introduced by electromagnetic networks and to enhance safety through electrical isolation, it is recommended to utilize the MADQ system’s battery power capability. Interferences, such as EMG components overlapping with ECG signals (Figure 11a), observed in this study due to improper placement of the reference electrode, can be also mitigated using additional signal-processing techniques. One approach is the application of narrower bandpass filters to reduce the interference; however, this approach may attenuate relevant ECG features. Alternatively, more advanced methods such as Independent Component Analysis (ICA) or adaptive filtering algorithms offer more targeted separation of overlapping sources without compromising signal integrity [60,61,62].

## 5. Conclusions

The MADQ system represents a significant advancement in flexible, research-grade physiological signal acquisition. It offers configurable multichannel capabilities (8–40 channels) across a broad sampling range (0.25–16 kHz) while maintaining low noise levels (0.24–1.7 µV RMS, ref. *f_s_*: 1 kHz), high Noise-Free Bits (NFB), and consistent Effective Number of Bits (ENOB) across varying gain settings. Its modular architecture enables true multimodal operation, supported by independent reference circuits. While certain performance constraints emerge at higher sampling frequencies, the MADQ remains well suited for most electrophysiological applications. Preliminary validation in simulated sleep studies, including auditory evoked potential detection, demonstrates MADQ’s potential for integration into multimodal experimental setups. However, further real-world testing is needed to fully assess its robustness across diverse experimental paradigms. The authors are currently planning follow-up studies where the MADQ system will be applied to more specific experimental contexts, such as a hybrid (EEG+EMG) brain–computer interface (BCI), multimodal evoked potential analysis, and sports science research. Ultimately, the MADQ system provides researchers with a powerful, customizable, and accessible tool for advancing electrophysiological studies and exploring novel paradigms for biomedical applications.

## Figures and Tables

**Table 1:** Maximum number of capture channels relative to *f_s_*.

Channel Type	Sampling Frequency—*f_s_* (kHz)				
Item 1	0.25, 0.5, 1	2	4	8	16
Electrophysiologic Channels	40	32	24	16	8
Digital GP Channels	4	4	4	4	4
Analog GP Channels	4	3	2	2	1

**Table 2:** Comparison between the MADQ and other signal acquisition systems.

System	# Channels	*f_s_* (kHz)	Noise (RMS)	Functional Features
MADQ	8–40	0.25 to 16.0	0.24–1.7 µV	Multimodal acquisition, high channel capacity, wide sampling range, low noise floor, customizable and free access.
OpenBCI Cyton	8	0.25	<1 µV	Wireless, compatible with open-source software, EEG-focused.
g.USBamp (g.tec)	16	Up to 38.4	<0.2 µV	High precision, proprietary software, medically certified.
NeXus Q32	32	Up to 4.0	<0.8 µV	Multimodal acquisition, portable and stationary setups, medically certified.

## Data Availability

Requests for access to the data supporting the results of this study be directed to the corresponding author via email (pcevallosl@ups.edu.ec).
